# 

**Published:** 1911-07-08

**Authors:** 


THE Year Book for 1911 issued by Livingstone College contains a useful review of recent literature on tropical medicine, and shows what excellent materials are available for those who desire to study these problems for themselves. Special attention is drawn to "The Prevention of Malaria," by Sir Donald Ross, and to the diagrams reproduced showing the rapid decrease of the mortality from malaria in Italy since the Italian Government started the distribution of quinine. The treatment of cholera by the use of permanganate of potash, as described by Major Leonard Rogers, is outlined, and many other advances in medicine are referred to. Among the achievements of old students may be noted the action of the Rev. E. W. T. Greenshields, who has been decorated by the Queen of Holland for rescuing the crew of a Dutch whaler, whilst in Assam the discovery of the *Culex pettigrewii* is to be credited to a former student. The financial statement shows a deficit of £246, which is a little less than that of the previous year, and this would probably have been wiped out had the number of students been maintained at the level reached two years ago.

### THE CHARITY ORGANISATION SOCIETY AND THE NATIONAL INSURANCE BILL.

The following resolution has been adopted by the Council of the London Charity Organisation Society: "That in view of the extremely complicated nature of the National Insurance Bill, the immense importance of the interests involved in it, and the fact that it seeks to introduce principles and methods entirely novel in the administration of this country, the results of which it is not easy to forecast, but which may in the gravest manner affect the well-being, character, and independence of the wage-earning classes, it is in the opinion of the Council of the Charity Organisation Society indispensable that the measure should not be pressed forward until such ample time has been given to inquiry and discussion as cannot possibly be secured in the present session of Parliament."

*To the Editor of* The Hospital.

### A Hospital President's Estate.

The Hon. Edward William Berkeley Portman, eldest son and heir of Viscount Portman, whose death and record of fourteen years' work as President of the Taunton and Somerset Hospital were published in The Hospital of May 6, page 147, has left estate of the gross value of £169,580, of which the net personalty has been sworn at £166,134.

### The Horton-Smith Prize.

The Raymond Horton-Smith prize, awarded annually for the best thesis presented to the University of Cambrige for the degree of M.D. during the academic year, has been won by Philip Hamill, M.A., M.D., Trinity College. Dr. Hamill is a St. Bartholomew's man, and is pathologist to the City of London Hospital for Diseases of the Chest, Victoria Park; his thesis was upon the mode of action of drugs. Dr. Comyns Berkeley was *proxime accessit* for the prize with a thesis upon malignant diseases of the uterus.

### THE QUESTION BOX.

### WHAT HOT-WATER BOTTLES LAST?

*To the Editor of* The Hospital.

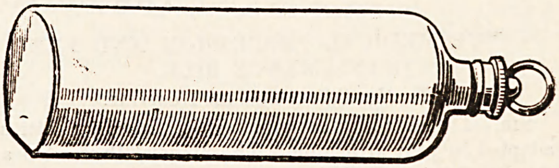

Sir,—In reply to a query in your columns *re* a suitable type of hot-water bottle, we have here adopted a new form to our own design. It is of "drawn" aluminium that is made from one piece of metal. It is bottle-shaped with a narrow neck. It is flattened at one side to prevent rolling. We subjected it to rigorous tests, such as partly filling with boiling water, screwing down firmly the brass top and allowing to cool. No tendency to collapse occurred. It was also dropped from a height so as to strike the floor first on the end, then on the side. The denting from this test was small, and we find dents easily made good. Its great lightness, freedom from rust, and easily maintained brightness of surface (dull finish) recommend it for hospital use. Perhaps its most important and least expected advantage is the length of time it retains its heat.

Soda or potash must not be brought into contact with it, and, of course, it requires to be well protected by flannel or other covering. It is made by Messrs. Jones Bros. & Co., Wolverhampton, and the design, I believe, is registered. The bottle is inexpensive.—Yours faithfully, J. S. Neil, House Governor Wolverhampton and Staffordshire General Hospital.

### COMING EVENTS OF THE WEEK.

[Communications for this column must be sent to 29 Southampton Street, Strand, before noon on Wednesday.]

Tuesday, July 18.—The laying of the foundation-stone of the extension of the Miller General Hospital by the Earl of Dartmouth, President, at 5 p.m.

Thursday, July 13.—His Excellency the Hon. Whitelaw Reid distributes the prizes to the students of the London Hospital in the Library of the Medical College, at 3 p.m.

### APPOINTMENTS VACANT.

Full particulars of these vacancies can be obtained on reference to our advertisement columns :

Cardiff Union.—Assistant Medical Officer.

Kensington & Fulham General Hospital.—Surgeon in charge of Ear, Nose, and Throat Department.

Leicester Infirmary.—Assistant House Surgeon.

North Devon Infirmary, Barnstaple.—House Surgeon.

Royal Albert Hospital, Devonport.—Assistant House Surgeon.

Royal Surrey County Hospital, Guildford.—Assistant House Surgeon.

Sheffield Royal Infirmary.—Seventh Resident Medical Officer.

Wolverhampton General Hospital.—House Surgeon.

Mr. Basil Hughes, M.A., M.B., B.C. Cantab., B.Sc. Lond., L.R.C.P. Lond., F.R.C.S. England, has been appointed resident surgical officer at Bradford Royal Infirmary.

